# Complication rates following all-epiphyseal ACL reconstructions in skeletally immature patients: A retrospective case series study

**DOI:** 10.1097/MD.0000000000027959

**Published:** 2021-11-24

**Authors:** Lydia Saad, Guy Grimard, Marie-Lyne Nault

**Affiliations:** a CHU Sainte-Justine, 3185 Chemin de la Côte Ste-Catherine, Montréal, Québec, Canada; b Université de Montréal, Montréal, Québec, Canada; c Hôpital du Sacré-Cœur de Montréal, Montréal, Québec, Canada.

**Keywords:** anterior cruciate ligament, all-epiphyseal technique, complications, pediatric

## Abstract

The aim was to evaluate the safety of a physeal-sparing anterior cruciate ligament reconstruction technique (ACLR), performed with Orthopediatrics (Warsaw, IN) equipment, by assessing complications.

Skeletally immature patients who underwent all-epiphyseal ACLR between 2015 and 2017 with postoperative follow-up were included in this retrospective study. Complications, demographic, clinical, surgical, and imaging data was retrieved from an urban tertiary pediatric hospital database. Physeal status, limb-length discrepancies (LLD), and angular deformities were assessed on preoperative and postoperative radiographs, growth disturbances were reported, and initial and follow-up diameters of tunnels were compared.

Nineteen ACLRs were included from 18 patients, 4 females and 14 males, with bone age at surgery of 13.3 ± 1.0 years. At a mean follow-up of 19.2 ± 10.1 months, there were no symptomatic growth disorders requiring intervention. There were: 2 (11.1%) unilateral early physeal closures, 2 (10.5%) new angular deformities (5°–10°), 4 (22.2%) LLD (1–2 cm), 1 (5.6%) contralateral ACLR, 1 (5.6%) femoral screw removal, 2 (10.5%) graft ruptures, and 1 meniscal tear (5.3%). Mean tunnel widening was 1.7 mm and 1.5 mm on the femoral and tibial side, respectively, and no massive osteolysis was recorded at the polyetheretherketone implant site.

The complication rates were comparable to those in similar studies, with no growth-related complications at 19.2 months.

## 1 Introduction

Anterior cruciate ligament (ACL) ruptures are increasingly diagnosed in the pediatric population^[1]^: diagnoses have increased 18.9% between 2007 and 2011 (in 10- to- 14-year-olds), according to a national database study.^[2]^ This might be the result of greater clinical awareness and better diagnostic methods,^[3]^ but could also be attributed to an actual increase in ACL tears, stemming from intense year-round training, competitive-level sports practice, and early sport specialization in young athletes.^[2–4]^ Conservative treatment is associated with poorer outcomes because of subsequent meniscal and chondral injury risks, especially in young patients with lower compliance.^[5,6]^ However, the optimal surgical technique is still debated.^[7]^ Standard transphyseal adult reconstruction involves a risk of growth disturbance in patients with open physes.^[8–10]^ Physeal-sparing alternatives were developed to avoid damage to the growth plates in pediatric patients with markedly open physes with an increased growth disturbance risk, even when transphyseal tunnels are drilled vertically and centered.^[11]^ These can be classified as partial transphyseal,^[12]^ all-epiphyseal,^[13]^ and extra-articular iliotibial band reconstructions.^[14]^ Although these surgeries are inherently designed to preserve the physes, limb-length discrepancies (LLD) and angular deformities were still observed in patients who underwent these procedures.^[15,16]^

The primary objective of this study was to evaluate the safety, with regards to potential growth disturbances, of the all-epiphyseal ACL reconstruction (ACLR) technique carried out with the first equipment tool kit specifically designed for pediatric patients. The primary outcome variables were limb-length discrepancies and angular deformities of the operated knee at the latest orthopedic follow-up. As a secondary objective, this study aimed to assess non-growth-related complication risks associated with the open physes technique and the equipment used in our data set. These non-growth-related complications included residual laxity rates, subsequent injury rates (graft rupture, meniscal tear, contralateral tear) and tunnel enlargement. The hypothesis was that this new equipment was safe, that is, that it would not produce growth complications that require corrective surgeries and that other rates of major complications would be comparable to those in the recent literature.

## 2 Methods

### 2.1 Ethics

This study was performed in line with the principles of the Declaration of Helsinki. Approval was granted by the Ethics Committee of the CHU Sainte-Justine review board committee (#2018–1703). Informed consent by participants was waived by the Ethics Committee.

### 2.2 Study design, setting, and sample size

This is a retrospective case series study. All of the twenty skeletally immature patients who underwent arthroscopic ACLR with the Orthopediatrics (Warsaw, IN) instrumentation between March 2015 and March 2017 at a large urban tertiary pediatric hospital were included in the primary group of patients. The surgery with Orthopediatrics equipment started being performed at this specific hospital in March 2015 and thus included all eligible patients who underwent it at the time of the data compilation. Sample size was not calculated pre-emptively.

### 2.3 Participants

From the original 20 patients who had the studied procedure, 2 patients were excluded because their follow-up was completed at another institution, and related data was unavailable. The final study group comprised 18 patients (19 knees) who were followed at the pediatric orthopedics clinic at the CHU Sainte-Justine. Two pediatric orthopedic surgeons specialized in sports medicine performed all surgeries jointly. The decision to perform an all-epiphyseal reconstruction procedure was based on the anterior tibial tuberosity status and the Greulich and Pyle bone age.^[11,17]^ Patients with an open physis (with a bone age under 12 years) and an anterior tibial tuberosity not sufficiently ossified to be viewed on radiographs for safe epiphyseal bone tunnel drilling, had a combined extra- and intra-articular IT band reconstruction. The others – those included in this study – underwent an all-epiphyseal reconstruction. All patients followed the institution's physical rehabilitation protocol.

### 2.4 Surgical technique

The sartorius fascia was opened at the *pes anserinus* level. The gracilis and semitendinosus were harvested with a tendon striper, cleaned, folded, sutured, and pretensioned. A diagnostic arthroscopy was conducted with standard portals. Meniscal repair or menisectomy was performed when required. ACL remnants were removed. The tibial tunnel was drilled with a 30° angle above the horizontal line using the dedicated drill guide under fluoroscopic and arthroscopic imaging. The femoral tunnel was drilled using the femoral tunnel drilling guide and under fluoroscopic guidance, with the exit point located at the level of the lateral condyle. The arthroscope was introduced in both tunnels to verify if the physes had been damaged. The Shiedloc (Orthopediatrics Corp., Warsaw, IN) sleeve was positioned in the femoral tunnel. The graft was then passed through the femoral and tibial tunnels under arthroscopic guidance and positioned on the titanium hook implant on the tibial side. The graft tensioner was positioned, and the knee underwent several flexion-extension cycles. The ACL was fixed at 30° of knee flexion with an interference screw on the femoral side. A special feature of this technique is the tibial implant used. It was located outside the tibia and had the theoretical advantage of maximizing the length of graft within the bone tunnels to reach a minimum of 15 mm of intra-tunnel graft length.^[18]^

### 2.5 Data collection and analysis

#### 2.5.1 Patient characteristics

Patient sex, laterality, chronological and bone age at time of surgery, mechanism of injury, knee history, clinical presentation of injury, surgical delay, and follow-up time were retrospectively collected from the hospital database. Magnetic resonance and operative reports were used to compile concomitant injuries and surgical procedures. Graft type and source, as well as initial bone tunnel sizes, were retrieved from operative protocols.

#### 2.5.2 Complications

Limb-length discrepancies and angular deformities were assessed on the patients’ radiographs. LLD was obtained from measurements from the top of the femoral head to the center of the ankle. They were done on preoperative and postoperative bilateral full-leg standing antero-posterior EOS (low-dose X-ray weight-bearing imaging system) radiographs.^[19]^ The preoperative radiographs used were the ones taken at the closest date before surgery. The latest follow-up radiographs with acceptable image quality were used for the postoperative measurements. A difference of 2 cm in leg length was defined as a clinically significant discrepancy on the basis of previous studies and according to the Pediatric Orthopedic Society of North America treatment guidelines.^[16,20,21]^ It was linked to the ACLR intervention if not present prior to surgery. Coronal plane knee angulations were measured on the same radiographs with the mechanical axis as reference.^[22]^ Varus and valgus malalignments of 5° or more that were not present prior to surgery were associated with the interventions and considered significant. This threshold, previously defined by Volpi et al,^[23]^ was established to factor in the measurement error between radiographs and normal tibiofemoral alignment, believed to be optimal within 3° of the mechanical axis.

All other complications were retrieved from the hospital databases.

#### 2.5.3 Femoral and tibial tunnel diameters

**Figure 1:**
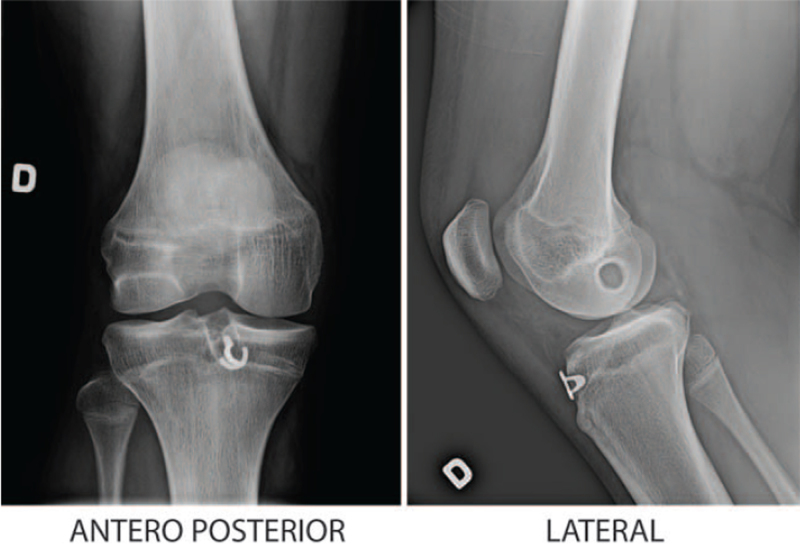
Radiographs of the largest tunnel enlargements measured after ACLR. The widest femoral tunnel diameter measurement was taken at the sclerotic margins of the bone. The same was done at the tibial tunnel. The Armorlink (Orthopediatrics Corp., Warsaw, IN) implant is visible at the tibia. Growth arrest lines are straight and well-defined, indicating no growth disturbance. Lateral left knee radiograph 13year-old boy with initial and final femoral tunnel sizes of, respectively, 7.5 mm and 11.2 mm. Anteroposterior left knee radiograph 13-year-old boy with initial and final tibial tunnel sizes of, respectively, 7.5 mm and 12 mm.

Femoral and tibial bone tunnel diameters were measured on postoperative lateral and antero-posterior knee radiographs, respectively (Figure 1). The widest tunnel measurements were taken using the sclerotic tunnel margins as reference points and compared to the known drill bit sizes retrieved from operative protocols. Those measurements could not be included in the statistical analysis because there was no calibration object on the radiographs, making it impossible to compare drilling size to measurements.

### 2.6 Analysis

Descriptive statistics were performed for demographical, surgical and growth characteristics with SPSS Statistics v25.0.

## 3 Results

Nineteen ACLRs (18 patients) with transepiphyseal tunnels were performed. All patients, except one, had autologous quadrupled hamstring tendon grafts. One reconstruction (5.3%) was done with a quadrupled hamstring graft augmented with gracilis allograft tissue. Average time from injury to surgery was 15.8 ± 15.2 months. There were 11 concomitant single lateral meniscal tears: 10 were sutured and one was partially resected with the ACLRs. There was one case of double meniscal lesions that required surgery, with the lateral meniscus undergoing partial resection and the medial meniscus undergoing repair. The remaining seven cases either did not present any meniscal lesion at the time of surgery, or had small lesions that did not require treatment or were rasped to help them heal. Patient demographical and surgical data is summarized in Table 1, while Table 2 summarizes growth characteristics.

**Table 1:** Presentation and treatment of ACL tears in skeletally immature patients.

Patient characteristics	Mean ± SD or N (%)
Chronological age (yrs)	13.5 ± 1.6 (10–16)
Bone age (yrs)	13.3 ± 1.0 (11–15.25)
Latest follow-up time (mo)	19.2 ± 10.1 (7–37)
Time between injury and surgery (mo)	15.8 ± 15.2 (2–58)
Patients	18
Knees	19
Sex	
Male	14 (77.8.0)
Female	4 (22.2)
Laterality	
Right	9 (47.4)
Left	10 (52.6)
Mechanism of injury	
Contact	4 (21.1)
Non-contact	14 (73.7)
Unavailable	1 (5.3)
Concomitant injuries/findings	
Lateral meniscus lesion	17 (89.5)
Medial meniscus tear	6 (31.63)
Lateral discoid meniscus	1 (5.3)
Bone contusion	5 (26.3)
Osteochondral lesion	4 (21.1)
Fracture	3 (15.8)
Popliteus and/or biceps femoris tendons trauma	1 (5.3)
LCL injury	3 (15.8)
MCL injury	2 (10.5)
PCL cane deformity	2 (5.3)
Surgical data	N (%)
Graft source	
Autograft	18 (94.7)
Hybrid	1 (5.3)
Graft type	
Hamstring (ST-G^∗^)	18 (94.7)
Hamstring and gracilis augment	1 (5.3)
Meniscal lesion management	
Lateral meniscus	17 (89.5)
Repair	10 (52.6)
Partial resection	2 (10.5)
Non-surgical or avivement only	5 (26.3)
Medial meniscus lesion	6 (31.6)
Repair	1 (5.3)
Non-surgical or rasping only	5 (26.3)

∗ ST-G = semitendinosus and gracilis quadrupled hamstring autograft.

**Table 2:** Preoperative and postoperative growth characteristics.

	Preoperative	Latest follow-up
	Mean or N (%)	Mean or N (%)
Physes status		
Open	19 (100.0)	12 (66.7)
Bilateral closure	0 (0.0)	5 (27.8)
Unilateral closure	0 (0.0)	2 (11.1)
LLD (mm)^∗^	6.1 ± 4.7 (0–15)	6.9 ± 4.7 (0–15)
Less than 1 cm	11 (57.9)	13 (68.4)
Between 1 and 2 cm	4 (21.1)	4 (21.1)
More than 2 cm	0 (0.0)	0 (0.0)
Unavailable	4 (21.1)	2 (10.5)
Angular deformity^∗∗^		
Injured knee (degrees)	−0.7 ± 2.3 (–6–3)	−0.6 ± 2.8 (–7–5)
Minor angulation (<5°)	14 (73.7)	15 (78.9)
Significant varus (≥5°)	0 (0.0)	1 (5.3)
Significant valgus (≥5°)	1 (5.3)	1 (5.3)
Unavailable	4 (21.1)	2 (10.5)
Contralateral knee (degrees)	0.3 ± 2.6 (–7–4)	0.7 ± 2.4 (–3–4)
Minor angulation (<5°)	14 (77.3)	17 (89.5)
Significant varus (≥5°)	0 (0)	0 (0.0)
Significant valgus (≥5°)	1 (5.3)	0 (0.0)
Unavailable	4 (21.1)	2 (10.5)
Harris growth arrest lines		
Femur	0 (0.0)	7 (36.8)
Tibia	0 (0.0)	7 (36.8)
Unavailable radiographs	0 (0.0)	1 (5.3)
Number of knees	0 (0.0)	9 (47.4)

∗ LLD = limb-length discrepancy.

∗∗ Negative angle values were used for knee valgum while positive angle values were used for knee varum.

**Figure 2:**
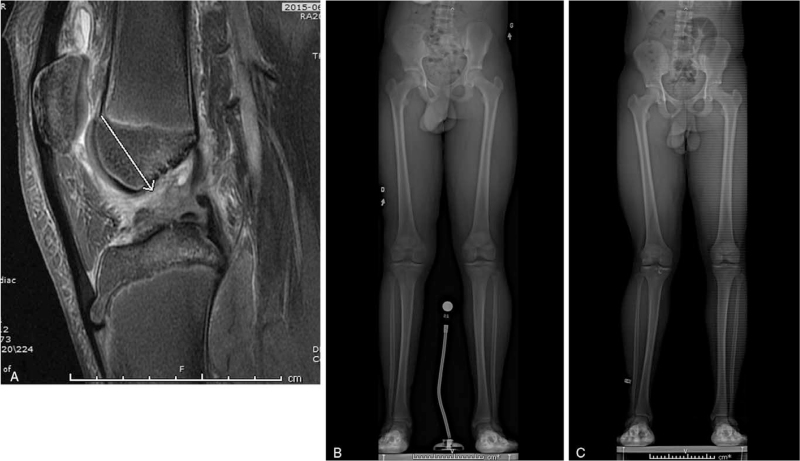
Preoperative MR imaging (a) and EOS radiograph (b) showing a 5 mm limb length discrepancy. At 3 years postoperative the EOS radiograph of the operated limb (c) shows 6 degrees of valgus.

Seventeen of the 18 patients had a bilateral lower-limb EOS radiograph at the latest follow-up. For the patient with missing imagery, there were no clinical growth disturbances noted at latest follow-up, and the patient was skeletally mature. There were no symptomatic growth abnormalities requiring an intervention among the patient cohort. One new isolated 11 mm overgrowth at the operated limb was observed in a skeletally mature patient at the latest follow-up. Another patient, also skeletally mature at the latest follow-up, had an isolated 6° knee valgus. The patient who underwent ACLR surgery on both knees had a last follow-up LLD of 9.5 mm. However, it was most likely not associated with the surgeries, as he had an LLD of 12 mm prior to the initial surgery. Another patient had a preoperative 6 knee valgus which was measured at 7° at the latest follow-up. We did not consider it a growth complication. However, 1 new isolated 5° varus angulation was observed. Overall, there was only 1 patient with an LLD reaching 1 to 2 cm or deteriorating after surgery (5.6% of patients), and 1 new angular deformity between 5 and 10° (5.3% of knees) (Fig. 2).

The transepiphyseal bone tunnels had a diameter between 6 and 8 mm at surgery, depending on the graft size. Average tunnel enlargement was 1.5 ± 1.2 mm at the femur, and 1.7 ± 1.2 mm at the tibia. The largest differences between initial and latest follow-up tunnel diameters were 3.7 mm and 4.5 mm, for the femoral and tibial tunnels, respectively, as shown in Table 3.

**Table 3:** Tunnel widening.

	Initial size Mean ± SD (range)	Latest follow-up size Mean ± SD (range)	Tunnel widening Mean ± SD (range)
Femoral tunnel	7.3 ± 0.5 (6–8)	9.0 ± 1.6 (6.1–11.5)	1.7 ± 1.4 (–0.9–3.8)
Tibial tunnel	7.1 ± 0.6 (6–8)	8.7 ± 1.4 (5.8–11.3)	1.5 ± 1.0 (–0.2–3.8)

The largest tunnel enlargements were noted in the same patient, who did not suffer complications at the latest follow-up. One patient (5.6% of patients) had chronic bilateral instability that required a contralateral ACLR procedure. There was 1 (5.3% of knees) case of femoral screw removal surgery in a patient with persistent bursitis on the lateral femoral side. This patient, as well as another patient, had a graft rupture, which brought the graft rupture rate to 10.5% (of knees). One (5.3% of knees) medial meniscus tear occurred 10 months after ACLR surgery. Other complications, not growth-related, are summarized in Table 4.

**Table 4:** Other complications following ACLR.

Complications	N (%)
Subsequent injuries	
Graft rupture	2 (10.5)
Contralateral ACL tear	1 (5.3)
Medial meniscus tear	1 (5.3)
Bursitis at femoral fixation site requiring hardware removal	1 (5.3)
Minor complications	3 (15.8)
Stitch abscess	1 (5.3)
Erythema	1 (5.3)
Temporary oedema at fixation site	1 (5.3)
Residual laxity	8 (42.1)
Inconclusive Lachman test	7 (36.8)
Inconclusive pivot-shift tests	5 (26.3)

## 4 Discussion

This study found that the all-epiphyseal reconstruction technique used in this group of pediatric patients had rates and types of growth complications comparable to those of other techniques.^[2,4,7,15,24,25]^ No clinically significant growth abnormalities had been observed yet, apart from 1 new unilateral angular deformity that did not require intervention and 1 limb overgrowth.

Positioning the transepiphyseal femoral tunnel at the center of the anatomic ACL footprint, located less than 3 mm from the growth plate,^[26]^ without damaging the femoral physis was challenging given the precision required. Animal studies showed that as little as 7 to 9% of physeal volume insult is sufficient to affect growth,^[27]^ which might have occurred in this group of patients despite the intra-operative arthroscopic findings that did not suggest major trauma to the physes.

Further damage to the physes might have occurred postoperatively, due to tunnel widening, as the 2 unilateral physeal closures in this series correlated with notable tunnel enlargement. Another explanation for mild growth disturbances is thermal damage to the physis, resulting from tunnel drilling. This hypothesis was suggested in a growth disturbance post- all-epiphyseal ACLR case report^[28]^ and described in an animal laboratory study.^[29]^ Lawrence et al also suggested that transepiphyseal tunnel drilling could have affected the blood supply to the physes on the epiphyseal side of the tibia and femur, thus creating minor damage to the growth plates.^[29]^ Another well-known etiology of growth disturbance is the presence of bone plugs through the physes, which can be harvested with bone–patellar tendon–bone grafts,^[26]^ an issue that was avoided in this series of patients through the use of hamstring tendons.

Bone tunnel enlargement following ACLR is a widely reported phenomenon.^[30,31]^ The bone tunnel widening associated with the Shieldloc and Armorlink (Orthopediatrics Corp., Warsaw, IN) implants used in the all-epiphyseal patients of this series had yet to be documented in recent literature. In this study, the tunnel enlargement recorded in the skeletally immature patients was more important than the ones previously reported in adult studies (maximum at femur: 1.8 mm, maximum at tibia: 1.7 mm).^[31,32]^ Tunnel enlargement in this pediatric population is proportionally greater, given the smaller dimensions of the knees.

The potential consequences of tunnel widening include growth disturbance,^[29,30]^ as previously noted, and residual laxity.^[33]^ The relationship between growth problems and tunnel widening was not assessed in a formal statistical model in this series of patients. However, the 2 patients with identified growth disturbances did not have enlarged tunnels, and neither did the patients with graft rupture. The femoral fixation device used in this study was made out of polyetheretherketone (PEEK), a nonabsorbable radiolucent thermoplastic polymer, which has not been associated with inflammatory responses.^[34]^ Significant tunnel widening has previously been associated with the PEEK Aperfix System (Cayenne Medical, Inc, Scottsdale, Arizona), although no clinical impact was found.^[35]^ Overall, the use of PEEK material for ACLR graft fixation showed good outcomes,^[36]^ but few studies report its use for ACLR. In this study, a persistent bursitis that required surgery in 1 patient, and a case of edema that self-resolved at femoral fixation site, suggest that there could be an increased risk of implant discomfort associated with PEEK. This remains a hypothesis, given the small sample size and the small rate of serious complications related to the femoral implant (5.3% of knees), which is similar to other recent studies (4.2%).^[8,37]^ A titanium alloy implant, rather than PEEK, was used for graft suspension at the tibia, where tunnel enlargement was also significant. However, it should be noted that many factors other than the materials used are known to contribute to this phenomenon: tunnel position, fixation method, bone quality, type of graft, etc.^[30]^ Moreover, the tibial tunnels were oblique in the lateral radiographs, and the manner in which they were measured – taking the largest diameter on the sagittal plane on non-calibrated radiographs –could have led to an overestimation of their sizes. It should be noted that the follow-up radiological measurements were compared to the tunnel sizes estimated from the drill bit sizes rather than from immediate postoperative X-rays. Measurement errors could therefore have impacted the results. Absolute differences, even though statistically significant, are less than 2 mm.

The short-term incidence of graft rupture (10.5% of knees) in this group of patients was acceptable, as it was close to rates reported in recent literature (8.7%).^[38]^ The tibial implant used was located outside the tibia with the theoretical advantage of maximizing graft length within bone tunnels, reducing the risk of re-tear.^[15]^ Further follow-up is needed to assess this complication because graft diameter shrinkage, after the one-year follow-up or more, increases the risk of re-tear.^[39]^ Indeed, decreased graft length is a well-known risk factor for graft tear.^[40]^ It should also be noted that graft rupture occurred in the only patient with a hybrid graft. Pennock et al recently found that the augmentation of small grafts with allograft tissue increases the risk of subsequent rupture.^[41]^ Contralateral tears have a low incidence in this study (5.6% of patients vs 8% in recent literature).^[25]^

This study is the first to report the outcome of pediatric ACLR performed with Orthopediatrics instrumentation regarding complications, especially regarding the peek femoral implant. Unlike other studies on pediatric ACLR complications,^[42]^ it included an assessment of preoperative growth abnormalities that was used to correlate LLDs and angular deformities with surgery. Another of its strengths is that patients underwent a standardized surgery, as it was performed by 2 surgeons who routinely work together.

Limitations of this study include the absence of sagittal knee angulation assessment. Furthermore, because of its retrospective nature, the quality of knee radiographs was sometimes poor. Also, initial tunnel sizes were derived from the sizes of the drill bits in the operative reports, which may have led to an overestimation of tunnel enlargement, as the insertion of ShieldLoc screw systems on the femoral side might have dilated the bone tunnels. These measurements would have been more accurate if immediate postoperative radiographs had been taken, to provide a comparative reference to the latest follow-up radiographs. Given the retrospective nature of this study, immediate postoperative radiographs were not available. However, initial sizes, as well as postoperative measurements, were still taken in the same manner for all patients. The sample was also small and heterogeneous in terms of surgical delay and follow-up time, as this series is composed of the first consecutive patients to undergo the surgery, which has only been performed starting from 2015.

## 5 Conclusions

This study found that all-epiphyseal reconstructions performed with Orthopediatrics equipment appeared to be a safe option in pediatric patients at a mean follow-up of 24.3 months. Short- to medium-term complication rates, regarding growth disturbance, graft rupture, and contralateral rupture, are comparable to similar studies. However, notable tunnel enlargement was observed in the distal femoral and proximal tibial epiphyses and is to be monitored closely.

## Author contributions

**Conceptualization:** Marie-Lyne Nault, Lydia Saad, Guy Grimard.

**Data curation:** Marie-Lyne Nault, Lydia Saad, Guy Grimard.

**Formal analysis:** Marie-Lyne Nault, Lydia Saad, Guy Grimard.

**Methodology:** Marie-Lyne Nault, Lydia Saad.

**Supervision:** Marie-Lyne Nault.

**Validation:** Marie-Lyne Nault.

**Writing – original draft:** Lydia Saad.

**Writing – review & editing:** Marie-Lyne Nault, Lydia Saad, Guy Grimard.
